# Tissue-Resident T Cells: Dynamic Players in Skin Immunity

**DOI:** 10.3389/fimmu.2014.00332

**Published:** 2014-07-16

**Authors:** Scott N. Mueller, Ali Zaid, Francis R. Carbone

**Affiliations:** ^1^Department of Microbiology and Immunology, Peter Doherty Institute for Infection and Immunity, The University of Melbourne, Parkville, VIC, Australia; ^2^The ARC Centre of Excellence in Advanced Molecular Imaging, The University of Melbourne, Parkville, VIC, Australia

**Keywords:** skin immunity, tissue-resident memory T cell, intravital imaging, two-photon microscopy, DETC, cell migration

## Abstract

The skin is a large and complex organ that acts as a critical barrier protecting the body from pathogens in the environment. Numerous heterogeneous populations of immune cells are found within skin, including some that remain resident and others that can enter and exit the skin as part of their migration program. Pathogen-specific CD8^+^ T cells that persist in the epidermis following infection are a unique population of memory cells with important roles in immune surveillance and protective responses to reinfection. How these tissue-resident memory T cells form in the skin, the signals controlling their persistence and behavior, and the mechanisms by which they mediate local recall responses are just beginning to be elucidated. Here, we discuss recent progress in understanding the roles of these skin-resident T cells and also highlight some of the key unanswered questions that need addressing.

## Immune Cell Subsets in Skin

The skin is a complex organ with critical roles in defense against pathogens. The epidermis forms a physical barrier that limits entry of microorganisms that make up the substantial microbiome on the skin (1), as well as pathogens and substances in the environment. The stratified layers of the epidermis are composed of specialized epithelial cells: the keratinocytes. The outermost layer of the epidermis, the stratum corneum, is composed of dead keratinocytes (corneocytes) that perform the main barrier functions. Keratinocytes in the basal layer of the epidermis are responsible for establishing the upper layers of the epidermis through cell division, and progeny of these cells migrate upwards as they differentiate and eventually die (2). Keratinocytes have key roles in immune defense via the production of cytokines, chemokines, and antimicrobial proteins in response to environmental or pathogenic stimuli. Cytokines and chemokines produced by keratinocytes alert cells in the dermis and in lymph nodes (LN) draining the skin of potential danger as well as recruit cells of the immune system (including neutrophils, monocytes, and T cells) to the skin.

The dermis is separated from the epidermis by a continuous basement membrane. The epidermis is interspersed with invaginations for hair follicles that are themselves, also lined by basement membrane and separated from the dermis. The dermis is composed of a network of fibroblasts that produce a collagen-rich extracellular matrix. In addition, blood vessels and lymphatic vessels are distributed throughout the dermis, facilitating entry of immune cells from the blood and exit to the LN, respectively.

**Figure 1:**
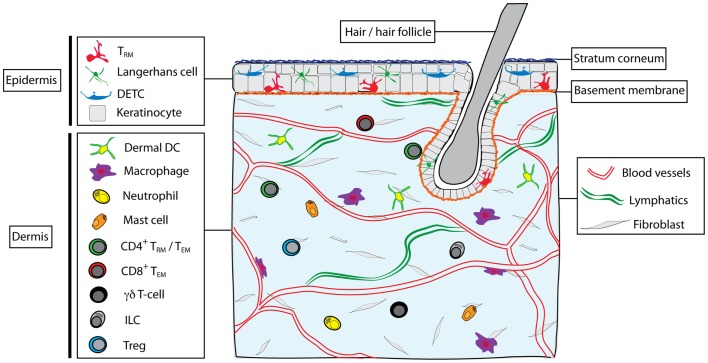
**Skin structure and immune cell types found in skin**. The skin is composed of epidermis and dermis, interspersed with hair follicles. Dead keratinocytes construct the stratum corneum in the outer epidermis. The dermis and epidermis are separated by a basement membrane. Blood vessels and lymphatic vessels and a network of fibroblasts are found in the dermis, as well as nerves, sebaceous glands, sweat glands (not shown). Multiple immune cells types are found within skin, including Langerhans cells, dendritic epidermal γδT cells (DETC), and memory αβT cells (T_RM_) in the epidermis. In the steady state, the dermis contains a heterogeneous mix of immune cells, including subsets of dendritic cells (including CD11b^+^ and CD103^+^ DC), macrophages (including dermal and perivascular macrophages), neutrophils, mast cells, γδT cells, ILC, CD4^+^ T cells (both T_EM_ and possibly T_RM_ subsets), T regulatory cells (Treg), and CD8^+^ T_EM_.

A variety of immune cells are present in normal skin (Figure 1), including subsets of dendritic cells (DC) and lymphocytes, as well as macrophages, mast cells, and neutrophils (3). DC resident in the dermis can be divided into two main subsets: CD103^+^CD11b^−^ (that can be further divided into langerin +/−) and CD11b^+^ (that can either be derived from steady-state precursors or from monocytes recruited during inflammation) (4). These DC migrate throughout the dermis before egressing via the afferent lymphatics to the LN where they either directly present antigen to T cells or transfer antigens to DC resident in the LN (5). In the epidermis, Langerhans cells (LC) form a dense network of DC capable of capturing antigen and migrating to the LN after traversing the basement membrane into the dermis. In mice, LC appear to be particularly efficient at tolerance induction and the formation of regulatory T cells (Tregs) (6, 7), whereas they are dispensable for induction of CD8 T cell responses to infections (8–10). Skin macrophages also populate the dermis and include perivascular macrophages that are distributed along post-capillary venules and can assist with the recruitment of neutrophils from the blood (11). Furthermore, a number of innate CD3^−^ lymphocytes (ILC) have been described in the skin, including NKp46^+^ ILC1 NK cells (12), CD90^hi^ ILC2 cells that produce IL-13 (13), and more recently NKp44^+^ ILC3 cells in human skin with psoriatic lesions (14). These ILC appear to reside in the dermis where they can interact with resident cells such as mast cells (13).

**Figure 2:**
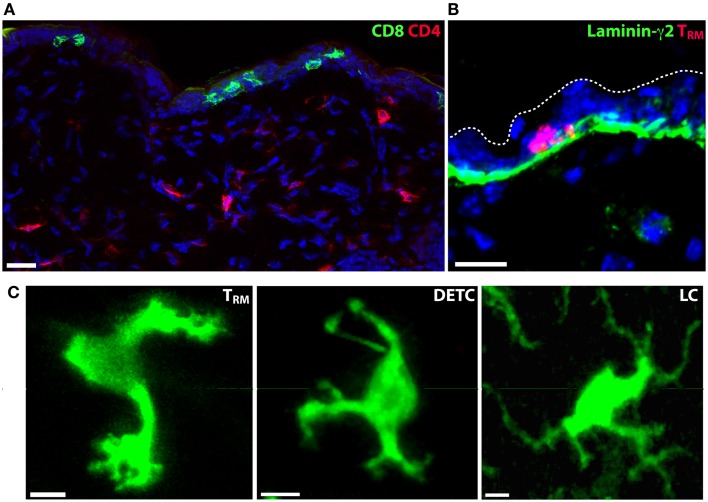
**Tissue-resident immune cells in the epidermis**. **(A)** CD4^+^ and CD8^+^ T cell localization in the skin of mice following clearance of HSV-1 infection. CD4^+^ T cells (red) localize to the dermis, while CD8^+^ T_RM_ persist in the epidermis. Nuclei are stained blue with DAPI. **(B)** Skin T_RM_ localize to the basal epidermis in contact with the basement membrane that separates dermis from epidermis. CD8^+^ T_RM_, red; laminin-γ2, green; DAPI, blue. **(C)** The morphology of epidermis-resident T_RM_, LC and DETC is distinct. Scale bars: A, B: 20 μm; C: 5 μm.

Lymphocytes are present in significant numbers in healthy skin, in particular CD4^+^ T cells, which populate the dermis (15). In contrast, B cells are rare in healthy skin. Tregs are also found in substantial numbers in healthy mouse dermis and their contribution to immunity or inflammation appears regulated by skin commensals (16). Skin Tregs display a much slower migrational velocity compared with effector CD4^+^ T cells although acute inflammation results in a rapid increase in their motility (17). CD8^+^ T cells are found in the skin in mice and humans, and are predominantly localized to the epidermis, in contrast to the predominantly dermal CD4^+^ T cells. This dichotomy is striking following herpes simplex virus infection (HSV) where antigen-specific CD4^+^ and CD8^+^ memory T cells localized to the dermis and epidermis, respectively, after clearance of the pathogen (18) (Figure 2A). The epidermal CD8^+^ T cells persist for long periods in this anatomical compartment (19) and are now commonly referred to as tissue-resident memory T cells (T_RM_).

Finally, populations of γδT cells are found in the dermis and epidermis, where they can contribute to wound healing and immune responses (20–22). Dendritic epidermal γδT cells (DETC) form a prominent network in the skin in mice where they appear to monitor the integrity of the epidermal layer. DETC form polarized immunological synapses that anchor at keratinocyte tight junctions (23). In response to infection or wounding, DETC upregulate molecules including NKG2D, JAML, and CD100 that contribute to inflammation and assist in wound closure (21, 24–26). DETC can also be infected by viruses such as HSV-1, which may influence their survival and functions during skin infections (27).

## Skin Tissue-Resident Memory T Cells

CD8^+^ T_RM_ cells that reside within the epidermis are retained in this compartment for very long periods without reentering the circulation (19, 28). Populations of T_RM_ have also been described in other tissues including the small intestine (29, 30), vaginal mucosa (18, 31), brain (32, 33), lung (34), salivary glands (35), and thymus (36). These cells can be identified by high expression of the α_E_ integrin chain (CD103) and the marker CD69 (19). T cells expressing this canonical T_RM_ phenotype have also been observed in other tissues such as the kidney, pancreas, and heart (32), while CD69^+^ T cells may also reside in LN and spleen for extended periods and provide a unique pool of cells that could guard against systemic pathogen entry (37).

Tissue-resident memory T cells that form in the skin, intestine, and lungs were recently shown to express a core set of genes that may facilitate accurate dissection of this memory T cell subset at a molecular level (38). This transcriptional signature suggests T_RM_ undergo a similar developmental program in different tissues. Elucidating the molecular pathways critical for T_RM_ development from tissue-derived signals will be important for future therapeutic approaches.

In addition to CD8^+^ T cells, some CD4^+^ T cells may also form a T_RM_ population in the lungs after respiratory viral infection (39). Although a proportion of the memory CD4^+^ T cells found within the dermis appear to be capable of entering the circulation (18), it is not yet clear whether the remaining cells permanently or semi-permanently reside in this site (i.e., could be designated T_RM_) and might thus be distinguished from circulating T effector memory cells (T_EM_).

During infection or inflammation of the skin, effector CD8^+^ T cells enter the dermis from the blood, and can then be recruited into the epidermis. This process is dependent on chemokine receptor signals, including CXCR3 (38). Whether other chemokine receptors are also required for CD8^+^ T cell entry into the epidermis is unclear, though this is likely since T_RM_ formation was only partially blocked when effector CD8^+^ T cells lacked CXCR3 expression. In contrast, in order to exit the skin via lymphatics T cells need to upregulate expression of CCR7, and blocking this step can promote increased T_RM_ formation. T_RM_ in skin develop from KLRG1^−^ effector cells that also give rise to classical central memory T cells (T_CM_) in the circulation (38). Once they enter the skin, these precursors migrate more effectively to CXCR3 ligands including CXCL10. Signals found within the epidermis instruct CD8^+^ T cells to develop into T_RM_ via upregulation of molecules involved in the persistence of these cells (CD103 and CD69), and downregulation of S1PR1 that is required for tissue egress (38, 40). Our recent data also revealed that T_RM_ express high levels of regulator of G protein signaling-1 (RGS1) and RGS2 (38). RGS1 expression has been shown to reduce T cell migration in response to CXCL12 and CCL19 (41), suggesting that these molecules may also contribute to the migration and persistence of T_RM_ within the skin as well as other tissues.

## Migration by Skin T_RM_

CD8^+^ T_RM_ localize to the basal layers of the epidermis in mice and appear to be in regular contact with the basement membrane that separates the epidermis from the dermis (42) (Figure 2B). Whether T_RM_ use this as a substrate for migration and adhesion is not known. Although the epidermal layer is considerably thinner in mice than in humans, CD8^+^ T cells also appear to localize to the border between the epidermis and the dermis in humans following HSV-2 infection (43) and in healthy or psoriatic skin (44). A unique feature of skin T_RM_ is their highly dynamic dendritic morphology (18, 42, 45) (Figure 2C). In contrast, T cells in the dermis consistently display a more amoeboid shape that is typical of T cells observed in all other tissues thus far. Whether T_RM_ in other tissues display a similar morphology and slow mode of migration is yet to be determined.

The immediate tissue environment appears to dictate the morphology and locomotion of T cells. This is supported by our observations in mice that both CD4^+^ and CD8^+^ T cells displayed a pronounced dendritic morphology when present within the epidermis, irrespective of whether the T cells were activated effector cells or memory cells (42). In addition, epidermal LC and DETC both adopt dendritic shapes. Notably, each of these cells (T_RM_, DETC, and LC) can be distinguished from each other by key differences in cell shape (Figure 2C). Whereas T_RM_ form many amorphous shapes marked by short dendrites and many small projections similar to filopodia, DETC produce a relatively consistent number of long dendrites and are mostly immotile because they are anchored in the upper epidermis. LC are immotile and produce multiple long, branched dendrites. While both DETC and LC project their dendrites upwards toward the stratum corneum, T_RM_ were only observed to extend projections laterally (42).

T cells migrating within the epidermis reduce their speed upon resolution of inflammation (42), suggesting that tight connections between keratinocytes present a difficult environment for T cells to navigate. It will be important to determine whether T_RM_ regulate unique molecules that facilitate digestion of the surrounding matrix and cell–cell adhesions to allow them to move relatively freely. The mechanisms used by T_RM_ to navigate the epidermis, including the molecules and pathways regulating the actin cytoskeleton to induce the unique cell shape are unclear. Since T cells migrating within tissues do not typically generate substantial protrusions such as lamellipodia or blebs (46), the way in which the actomyosin network generates force to propel T cells in the epidermis may differ from that in the dermis and other tissues. Moreover, the roles of adhesion molecules such as integrins and chemotactic factors including chemokines in controlling T cell migration in the epidermis is not known. The integrin CD103 is involved in the attachment of DETC dendrites to the keratinocytes (23). We found that CD103 expression by T_RM_ is important for their long-term retention in skin (38). This is unlikely to involve stable dendrite attachment due to the motile nature of T_RM_, though persistent adhesion to the keratinocytes via E-cadherin may facilitate the retention of T_RM_ in this site. T_RM_ also show increased expression of E-cadherin, the ligand for integrin α_E_β_7_, as well as the integrin α_1_β_1_ that binds collagen and laminin, both major components of the basement membrane separating epidermis from dermis. T_RM_ in skin have increased expression of the chemokine receptor CCR8 compared with memory T cells in other tissues (38). Expression of CCR8 is programed by the epidermis (47) suggesting that expression of this receptor is important for αβT cell residence in this site. Together, these receptors potentially contribute to adhesion, morphology, and survival of T cells in the epidermis.

## Immunosurveillance and Local Persistence of Skin T_RM_

Although T_RM_ localization in the skin can be relatively dispersed (28), they predominate at sites of infection or inflammation (19). This concentration of memory cells in mouse skin remains remarkably constant for >1 year after infection, despite their sustained motility (42). *In silico* simulation of the migration of T_RM_ in the skin over long periods revealed that T_RM_ move by random brownian motion and persist within the region of the epidermis in which they form simply as a result of this slow migration. These experiments suggest that T_RM_ induced by infection or vaccination should persist for very long periods in the immediate environment where they were formed and provide robust site-specific immunity. While such site-specific immunity may be of little use against subsequent infections at remote sites, repeated infections can induce T_RM_ in non-involved regions of skin (28), potentially providing more widespread protection at least in this tissue. Whether this is the case with many infections or tissues and the protective efficacy of these more dispersed T_RM_ needs to be investigated further.

Skin T_RM_ display a persistent mode of random migration that can facilitate surveillance of skin against reinfection or the recrudescence of latent viruses such as HSV (42, 45). Although T_RM_ migrate considerably slower in the epidermis of mice than T cells in the dermis or in lymphoid tissues, the cells are trapped within the constrained epidermal environment and move largely two-dimensionally. How T_RM_ survey the epidermis in humans remains to be visualized, although the location of these cells in the basal epidermis in human skin samples suggests that the mechanism and efficiency of immunosurveillance may be very similar to that observed in mice. Importantly, in addition to the shape and motility of T_RM_, the density of cells present in the epidermis will likely influence the efficiency of their surveillance, as suggested in experiments modeling T_RM_ migration (45). Therefore, novel vaccine strategies designed to induce T_RM_ in the skin or other sites in the body may need to reach a certain threshold of T_RM_ density in the tissues for effective protection against disease.

## Epidermal Niche

As mentioned above, large numbers of γδT cells (DETC) exist in the epidermis in mice, where they contribute to homeostasis, would repair and inflammation. In humans, γδT cells are present in the epidermis, though in lower numbers than αβT cells. The reason for this difference is unclear, though both T cell subtypes present in human epidermis can contribute to wound repair (21), suggesting that this may reflect a functional specialization of all T cells that persist in this tissue, as opposed to only γδT cells. DETC are the first T cells that form in mice very early in life. After migrating to the skin, they persist for life and are maintained by homeostatic turnover. Examination of DETC in mouse skin after the clearance of HSV infection revealed a substantial and sustained decrease in DETC numbers around the site of infection, and a corresponding increase in numbers of virus-specific T_RM_ (42). This inverse relationship between DETC and T_RM_ was maintained for months, suggesting that DETC were unable to repopulate regions of skin containing considerable numbers of T_RM_. These findings indicate the existence of a T cell-specific niche within the epidermis that regulates the total number of T cells in this site, irrespective of TCR usage or specificity. Both DETC and T_RM_ rely upon the cytokine IL-15 and signals via the aryl hydrocarbon receptor (AhR) for persistence in the skin (38, 42, 48, 49). AhR is a transcription factor that can regulate a large number of genes, including c-kit and various cell cycle genes, suggesting that this pathway may influence T cell proliferation and homeostasis in the epidermis (50). Ligands for AhR are produced in the epidermis via metabolism of tryptophan or from microbiota such as yeast. Nevertheless, AhR ligands are abundant in the skin, suggesting that other mechanisms likely also contribute to the regulation of T cell numbers in the epidermis.

If the epidermis constitutes a privileged niche with limited space for populations of T cells, this may have implications for T_RM_ persistence following subsequent infection or inflammation where new populations of effector CD8^+^ T cells are recruited to the skin. Therefore, whether there is a maximum number of T cells capable of persisting in the epidermal niche remains a key unanswered question. If so, we would expect that competition for space in this niche would restrict numbers of T_RM_ that can persist in regions of skin prone to multiple infections. Moreover, if effective protection from infection requires a certain density of T_RM_ in skin to rapidly respond, then competition for niche may influence such immunity. This also raises the intriguing question of whether low numbers of γδT cells in the epidermis of adult humans, and correspondingly higher numbers of αβT cells is, at least in part, the result of replacement of DETC via competition for space by T_RM_ that are generated by infections and environmental antigens. Developing a better understanding of the mechanisms of T cell homeostasis within the epidermis is critical for the design of strategies to boost immunity to infections as well as potentially reducing unwanted T cell responses.

## Protection by Skin T_RM_

Reinfection with a previously encountered pathogen results in recruitment of circulating memory T cells to the inflamed tissues where they function to eradicate the infection. CD8^+^ T cells recruited to tissues then clear the pathogen by killing infected cells and releasing cytokines. This process is still relatively slow, yet appears to be significantly enhanced by the presence of T_RM_ within the infected tissues. Notably, T_RM_ present within mucosal tissue epithelia were found to rapidly produce interferon-γ upon peptide stimulation, resulting in the non-specific recruitment of circulating memory T cells into the tissue within hours (51). Thus, it has been suggested that T_RM_ function as an antigen-specific sensor and rapidly respond by producing signals that induce local inflammation and recruit memory T cells from the blood. Though it is not yet clear whether T_RM_ in different tissues behave the same way, it will be important to determine what signals are released by T_RM_ in response to stimulation and the effects that these have on the subsequent response. For example, we found that T_RM_ express high levels of the chemokine XCL1, which may allow them to recruit XCR1^+^ cells such as dermal CD103^+^ DC (52) and facilitate local recall responses.

In addition to an alarm function, T_RM_ presumably also contribute directly to the clearance of pathogens in tissues. Whether they do this via the killing of target cells or production or cytokines, or both, has not yet been determined. Moreover, the relative contribution of T_RM_ versus memory T cells recruited from the circulation is not known. Thus, examination of whether skin T_RM_ have the capacity to eradicate a local infection without the assistance of circulating memory CD8^+^ T cells will provide insight into the role of resident memory in protective immunity. The relative roles of T_RM_ in raising the alarm versus directly clearing an infection may be influenced by their density with the tissue. We would predict that a high local density of T_RM_ could protect against viral infection and possibly provide sterile immunity. There is some evidence to suggest that T_RM_ also proliferate locally in response to challenge (53, 54), although the extent and widespread nature of this proliferation remains far from clear. Finally, given the restricted localization of T_RM_ to epithelial layers such as the skin epidermis, we might predict that these memory cells are terminally differentiated and highly dependent on their environment to survive. Experiments suggest this is the case, since isolation of T_RM_ from the brain followed by adoptive transfer into mice demonstrated poor survival and responses to challenge (33). Nevertheless, it remains unclear whether T_RM_ can exit the epithelial layers upon recall and migrate through tissues or enter the circulation, and if they do whether they can survive. Finally, experiments are needed to determine if T_RM_ form secondary memory in the tissues following restimulation, or are replaced by memory cells recruited from the circulation.

## Perspectives

There is considerable complexity in the immune cell content of the skin. While this content includes populations such as T cells and DCs, it is now clear that these are heterogeneous, comprised of a number of phenotypically and functionally distinct subsets. In the case of T cells in particular, their action is predominantly local, affording regional protection against skin-invading pathogens or promoting tissue repair after injury. Given the need for such restricted action, it is not surprising that the skin contains skin-resident populations. Despite this, the relative contribution of resident versus migrating cells still remains unclear in many instances. The existence of such uncertainty highlights the need for clear demarcation between resident and migrating populations in future studies of the skin immune system.

## Conflict of Interest Statement

The authors declare that the research was conducted in the absence of any commercial or financial relationships that could be construed as a potential conflict of interest.
